# Molecular and Structural Evolution of Cytochrome P450 Aromatase

**DOI:** 10.3390/ijms22020631

**Published:** 2021-01-10

**Authors:** Giovanna Di Nardo, Chao Zhang, Anna Giulia Marcelli, Gianfranco Gilardi

**Affiliations:** Department of Life Sciences and Systems Biology, University of Torino, via Accademia Albertina 13, 1023 Torino, Italy; chao.zhang@unito.it (C.Z.); anna.marcelli@edu.unito.it (A.G.M.)

**Keywords:** cytochrome P450, aromatase, estrogens, molecular evolution, structural alignment, substrate recognition sites, conservation

## Abstract

Aromatase is the cytochrome P450 enzyme converting androgens into estrogen in the last phase of steroidogenesis. As estrogens are crucial in reproductive biology, aromatase is found in vertebrates and the invertebrates of the genus *Branchiostoma*, where it carries out the aromatization reaction of the A-ring of androgens that produces estrogens. Here, we investigate the molecular evolution of this unique and highly substrate-selective enzyme by means of structural, sequence alignment, and homology modeling, shedding light on its key role in species conservation. The alignments led to the identification of a core structure that, together with key and unique amino acids located in the active site and the substrate recognition sites, has been well conserved during evolution. Structural analysis shows what their roles are and the reason why they have been preserved. Moreover, the residues involved in the interaction with the redox partner and some phosphorylation sites appeared late during evolution. These data reveal how highly substrate-selective cytochrome P450 has evolved, indicating that the driving forces for evolution have been the optimization of the interaction with the redox partner and the introduction of phosphorylation sites that give the possibility of modulating its activity in a rapid way.

## 1. Introduction

Aromatase is the enzyme that converts androgens into estrogens through a three-step reaction that allows the aromatization of the A-ring of the steroid molecule [1,2]. The enzyme belongs to the cytochrome P450 (P450s) superfamily that comprises thousands of enzymes involved in the metabolism of endogenous and exogenous substrates [3,4,5]. The origin of such a large number of enzymes is still controversial, even though the presence of a common ancient precursor, CYP51 (lanosterol 14alpha-demethylase), for both prokaryotes and eukaryotes has been hypothesized [6].

The P450 superfamily is composed of two groups of enzymes. Depending on their substrate recognition abilities, one group comprises P450s that catalyze specific reactions on specific endogenous substrates; a second group includes enzymes that have evolved towards broad substrate selectivity, usually employed for xenobiotic metabolism, as in the case of mammalian liver proteins. While for the second group, it can be hypothesized that evolution has widened their substrate selectivity, for the first one, it is not clear how molecular evolution has worked.

Aromatase belongs to the first group as it carries out the conversion of androgens into estrogens across different classes of living organisms. From an evolutionary point of view, its gene and activity have been found in invertebrates of the genus *Branchiostoma*, belonging to cephalochordates [7]. Indeed, aromatase, together with other P450 enzymes involved in steroidogenesis, have been found in the gonads of the invertebrate *Branchiostoma belcheri*, which is considered to be evolutionarily closer to vertebrates than other invertebrates [8,9].

The enzyme is present in all vertebrates as the product of expression of a single gene, with some exceptions represented by pigs and teleosts, where duplication events have produced three and two isoforms, respectively [10,11,12,13]. Furthermore, the protein is expressed in different tissues in vertebrates, where it plays an essential role in reproductive biology as estrogens are responsible for ovarian differentiation, development of the reproductive system, sex differentiation, and reproduction [14]. Moreover, a critical role of estrogens has also been demonstrated in brain, bone, skin, fat, and cardiovascular tissues [15,16,17,18,19,20]. In humans, tissue-specific regulation of aromatase gene expression is allowed by the presence of eleven promoters and alternative first exons [21]. However, a wide tissue distribution of the aromatase protein and a complex regulatory region in its gene is already present in fishes [22].

Vertebrates have been used as models to understand the roles of aromatase and estrogens in the different tissues where it is expressed. For example, in birds and mammals, it has been demonstrated that in the brain, there is a rapid modulation of aromatase activity through phosphorylation and that estrogens can be considered neurotransmitters [23]. Moreover, estrogens are involved in different processes, such as neurogenesis, neuroprotection, and cognition [22,24]. 

In reptiles and amphibians, temperature regulates aromatase expression and is responsible for temperature-dependent sex determination [25,26,27]. In some hermaphrodite fishes, sex changes occur in response to environmental cues related to social interactions, and aromatase is involved in the remodeling of the gonads during this process [28,29]. Due to the phenotypic effects as a consequence of androgen/estrogen unbalance, amphibians and fishes are widely used as model organisms to understand the possible effect of many compounds that also target human aromatase [30,31], known as endocrine-disrupting chemicals (EDCs) [32,33].

Among fishes, teleosts represent the only case where two isoforms are present (CYP19A1 and CYP19B1), and they are preferentially expressed in the gonads and brain, respectively. Interestingly, these isoforms have also been reported to have different catalytic activity in comparison to the human enzyme [34,35], indicating that functional differences can be present. Thus, it is interesting to understand the phylogenetic origins of these differences.

In this work, comparative sequence and structural analysis are used to investigate if and how the substrate-selective nature of aromatase has evolved, both in structural and functional terms. Its highly substrate-selective nature, calibrated for catalysis on androgens, makes it an optimal candidate for evolutionary studies, with the aim of (1) understanding if and how molecular evolution has structurally optimized this enzyme in order to make it more efficient and (2) determining what the conserved structural scaffold is and which are the amino acids that are essential for its function. Moreover, by identifying the functional amino acids that have not changed during evolution and excluding the ones shared with the other P450s, it is possible to obtain the fingerprint sequences of this enzyme. Structural analysis also allows us to identify a possible role for these residues and the rational basis for conservation. The most different aromatase sequences were also subjected to homology modeling to visualize where evolution has structurally modified the enzyme.

## 2. Results

### 2.1. Multiple Sequence Alignment 

#### 2.1.1. Structural Conservation

In order to identify the most conserved structural elements in aromatase, 365 sequences, ranging from invertebrates to mammals, were used for multiple sequence alignment. Out of the 365 sequences aligned, 66 were from mammals, 8 from birds, 12 from reptiles, 18 from amphibians, 259 from fishes, and 2 from the invertebrates of the genus *Branchiostoma*. 

For all the analyses performed in this work, the residue numbers refer to the sequence of human aromatase (CYP19A1, Uniprot ID P11511). 

**Figure 1:**
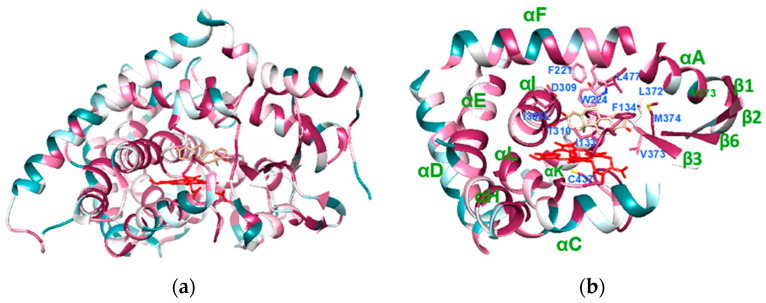
Crystal structure of human aromatase (PDB ID 4KQ8), colored according to the conservation. The violet areas correspond to the more conserved regions, whereas the dark green ones correspond to the most variable. Heme is shown in red and the substrate androstenedione in light brown. (**a**) Overall structure of human aromatase. (**b**) The core structure of aromatase, carrying the most conserved regions. The residues important for substrate binding are also shown.

When the positions of the most conserved regions were analysed in the crystal structure of the human enzyme, they resulted as part of helix A (65–78), the β-sheet formed by strands β1 (83–88) and β2 (93–97), helix E (187–205), part of helix F (221–224), the central part of helix I (residues 302–318 in human aromatase), helix K (354–366), the K-β3 loop and the β3 strand (368–376), the β6 strand (393–396), and helix L and part of the L-K’’ loop (427–448) (Figure 1). Helices C, D, F, and H carry conserved amino acids oriented toward the core of the protein and nonconserved amino acids exposed to the solvent. Thus, the conserved structural core in cytochrome P450 is formed by a four-helix bundle formed by helices D, E, I, and L that is conserved among aromatase sequences; an exception is made for the residues of helix D, exposed to the solvent (Figure 1) [36]. Helix G is not conserved, whereas the F-G loop and the first part of helix F, known to be important for opening the access channel in cytochrome P450, are conserved.

The key cysteine residue coordinating heme iron is obviously conserved in all the sequences, and it is within a consensus sequence formed by FGFGPRX_1_CX_2_GK/R, where X_1_ is variable (G, A, S, T, or N) whereas X_2_ is A, V, L, or I. This consensus sequence is also well-conserved in cytochrome P450 (FXXGX(H/R)XCXG), together with the meander region, a loop preceding the cysteine residue [36], which is also well-conserved in most aromatase sequences.

The three Arg residues involved in salt bridges with heme propionyl groups (R115, R145, and R435 in human aromatase) are also present in all the sequences, together with Trp141, and are involved in an H-bond with the heme propionyl group.

A highly conserved motif in cytochrome P450 is the EX_1_X_2_R motif located in helix K and involved in salt bridge interactions that are important for its tertiary structure and the correct incorporation of the heme cofactor [36]. This motif is conserved in all sequences; X_1_ is a serine residue, whereas X_2_ is L or M in most aromatase sequences.

#### 2.1.2. Functional Conservation

The level of conservation of amino acids that are relevant for substrate binding and catalysis was then verified in the multiple alignments. A highly conserved alcohol–acid pair is present on helix I in cytochrome P450, and it is part of the proton relay network that allows the formation of the reactive intermediate (Compound I) in the catalytic cycle. In aromatase, the alcohol–acid pair is formed by an aspartic acid residue (D309 in human aromatase) and a threonine residue (T310) that are conserved (exception is made for two fish sequences), and they are preceded by a proline residue (P308) in all the sequences analyzed. When compared to other P450s, this proline residue is unique to aromatase, and it is responsible for the shift of the I-helix axis observed in the crystal structure of the human enzyme [37]. Such a shift is important as it allows the 3-keto moiety of the substrate androstenedione to be accommodated near the fifth turn of the I-helix that is formed by M303 and A307. These two residues are conserved, with some exceptions. The methionine is substituted by an isoleucine in five fish sequences, one amphibian sequence, and one mammal sequence; there is an alanine residue that is a glycine residue in 4.5% of fish sequences and in two invertebrates. Moreover, the shift of the I-helix allows the formation of a hydrogen bond between D309 and the 3-keto oxygen of the substrate. Such an aspartic acid residue has never been changed into a glutamic acid during evolution due to its important role in substrate binding and catalysis [38]. All these residues (303–310) are located on helix I, and they are part of one of six substrate recognition sites (SRSs), namely, SRS-4. The residues involved in androstenedione binding are highly conserved, with some exceptions represented by few fish sequences (Table 1).

Two other residues are important for aromatase function; they are predicted to be part of the proton relay network that allows the formation of the reactive Compound I in the typical P450 catalytic cycle: R192 and E483. These residues form a salt bridge in the same position as the one found in the crystal structure of the bacterial cytochrome P450cam [39]. The residues R192 and E483 are highly conserved, starting from the sequences of aromatase from invertebrates. The crystal structure of the bacterial camphor-hydroxylating P450cam from *Pseudomonas putida* shows that this salt bridge is broken when the P450cam interacts with the redox partner that stabilizes the open conformation of the enzyme, exerting an effector role [39,40,41]. For human aromatase, the redox partner cytochrome P450 reductase (CPR) has been shown to promote substrate binding, acting as an effector [42], and the presence of the R192-E483 salt bridge in the same structural position as P450cam suggests that a similar effect can be exerted by its redox partner CPR.

#### 2.1.3. Conservation of the Substrate Recognition Sites (SRSs)

Six regions have been identified to be important for substrate recognition and binding in P450s: these are the so-called substrate recognition sites (SRSs). They are considered to be the most variable regions among cytochrome P450 as their variation during evolution is associated with new substrate selectivity. According to this idea, it is expected that the SRSs of aromatase, a nonpromiscuous enzyme that is highly selective for androgen substrates, have been highly conserved during evolution. Thus, the level of conservation of the six SRSs was checked and is shown in Table S1. As it can be seen, SRS-4 is the most highly conserved one (69.7% of the amino acids are conserved) as it carries amino acids crucial for catalysis, whereas SRS-3 has been highly variable during aromatase evolution (15.4% of conserved amino acids). In the other SRSs, about 40% of the amino acids are conserved.

As mentioned before, some residues in SRSs are shared in all P450s as they are essential for their catalysis. For example, in SRS-4, the acid–alcohol pair is not unique for aromatase as it is part of the proton relay network that allows the formation of reactive intermediates. Thus, in order to identify the residues that are conserved and unique for aromatase in the SRSs, multiple structural alignments of the 57 human P450s were performed using the server PROMALS3D. For structural alignment, the server uses the crystal structures available; their PDB IDs are used as input. When the structures are not available, the input sequences are aligned after secondary structure prediction, and 3D structure constraints are assigned based on homolog structures [43]. The multiple alignments obtained were then evaluated by the ConSurf server to assign a conservation score for each amino acid position.

Table S2 shows the residues belonging to the six SRSs in aromatase and the corresponding conservation score obtained from the alignment of the 363 sequences analyzed. Moreover, it shows the conservation score for the same positions obtained from the alignment with all the other human P450s. This comparison was performed to identify the residues conserved in the SRSs of all the human enzymes (shown in green in Table S2) and the ones specific for aromatase (shown in red in Table S2).

**Figure 2:**
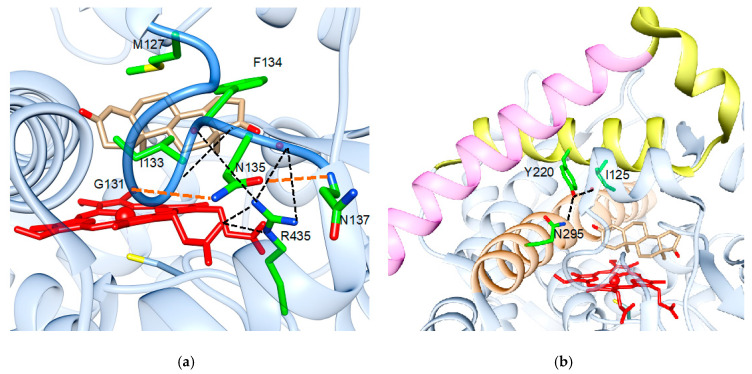
Role of the highly conserved residues in human aromatase (PDB ID 4KQ8). (**a**) Involvement of the highly conserved N135 in bridging G131 and N137 via H-bonds (shown in orange), which is important to maintain the B-C loop (blue) conformation and provide M127 and F134 to the active site of the protein. The H-bond network is shown in black. (**b**) Involvement of the highly conserved Y220 in H-bonds that connect N295 and I125. N295 is part of SRS-4, shown in orange, Y220 is part of SRS-2, shown in yellow, and SRS-3 is shown in magenta.

In SRS-1, helix C carries a Trp residue (W141 in aromatase) that is an aromatic amino acid in all P450s, important for heme binding. In many of them, it is followed by a positively charged residue (present in all CYP2, CYP3, and CYP26 members). R145 is conserved in most P450s as it is involved in heme binding, and the last two residues are small hydrophobics in many of them. K150/A151 are conserved and specific for aromatase. The helix B region is highly variable in human P450s. In aromatase, M127 is conserved as it delineates the active site cavity, whereas N135 is part of an H-bond network also involving R435, important for heme binding. The role of N135 is important as it bridges G131 and N137, keeping the B-C loop in a conformation that allows the highly conserved I133 and F134 to be part of the active site and to contact the substrate (Figure 2a).

SRS-2 is highly variable in P450s, and it carries conserved residues in aromatase. They are located on helix F and on the F-G loop. They are important flexible elements in P450s, including aromatase [44], as they are involved in the conformational changes that allow ligand access to the active site [45]. Out of them, Tyr220 forms an important H-bond with N295 that is part of SRS-4 and, with I125, defines the substrate access channel (Figure 2b).

In SRS-3, the cluster of three basic residues is not specific for aromatase as it is present in all CYP4F members, CYP46A1, and, within the same helix (helix G), CYP51. Interestingly, a glutamic acid is present before the cluster in all CYP4F members. EK is also present in some CYP26/27 members. Interesting, all these P450 families are involved in steroid, leukotriene, and retinoic and fatty acid processing [46,47,48,49,50].

SRS-4 and SRS-5 are the most conserved in human P450s. However, there are residues specific for aromatase, including I305 and M374, that are involved in substrate binding. In SRS5, the consensus sequence XEXXR is well conserved.

SRS-6 carries two His residues that are conserved in aromatase sequences that are part of a β-hairpin, whereas the other residues are not conserved.

#### 2.1.4. Consensus Sequence for Post-Translational Modifications

Post-translational modifications on human aromatase have been reported to alter its activity [51,52,53,54].

**Figure 3:**
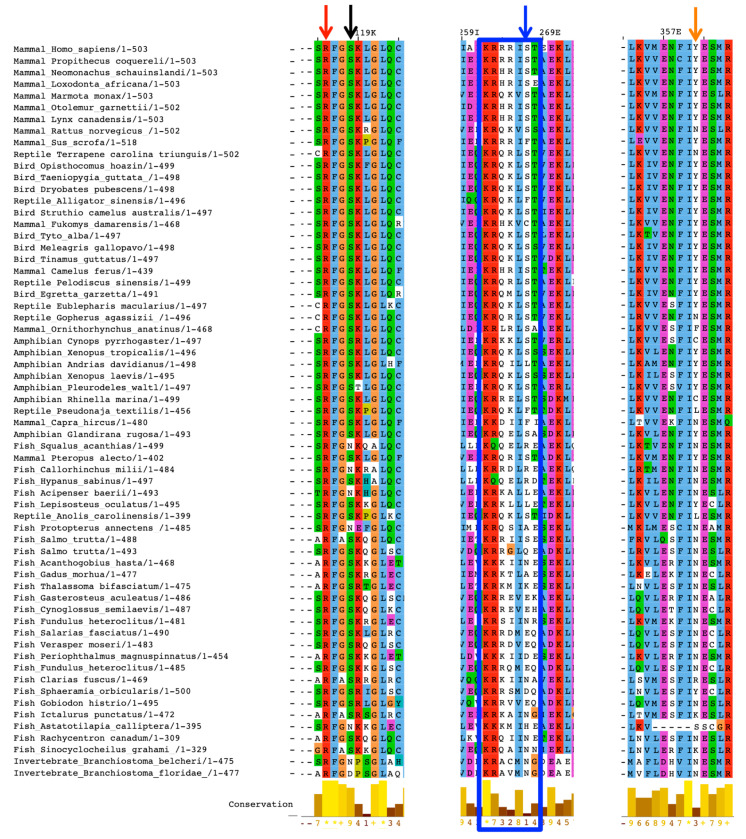
Multiple sequence alignments showing only representative aromatase sequences from the different classes of vertebrates and the two invertebrates. The three regions shown are the ones carrying the phosphorylation sites: S118 is indicated by the black arrow, and R116, which forms the consensus for PKA, is indicated by the red arrow. S267 is indicated by the blue arrow, and the cluster KRRIST, present on human aromatase, is shown in the blue box. Y361 is indicated by the orange arrow.

The region between amino acids 262 and 268 is a consensus sequence for different kinases such as PKA (R-X_1–2_-S/T-X) and PKG ((R/K)_2–3_-X-S/T-X). In human aromatase, the sequence is KRRRIST, where a cluster of four basic residues gives a positively charged patch on the surface that can attract opposite charges. However, only K and the first R are highly conserved, whereas the S and T residues are not conserved in fishes and the two invertebrate sequences (Figure 3). This means that the consensus sequence for PKA is present starting from amphibians. On the other hand, the consensus for PKG that includes two or three basic residues is present in only 15% of mammal aromatase sequences.

The other residue reported to be phosphorylated is S118, which is very well conserved, together with an arginine residue presenting two amino acids before (R116). The only exceptions are represented by six aromatase sequences from fishes and the two from invertebrates where serine is substituted by N or D (Figure 3). Thus, this consensus sequence for PKA is present starting from vertebrates.

The other important residue known to be phosphorylated is Y361. This residue is present in most mammal sequences (83%) and appears in amphibians, where it is present in 75% of the sequences. In mammals, where it is not present, it is substituted by N, as in most fishes, where a tyrosine residue is found only in 2.8% of the sequences analyzed (Figure 3).

#### 2.1.5. Interaction with the Redox Partner

The interaction of P450s with their redox partner is crucial for their function and catalytic efficiency. The docking site of cytochrome P450 reductase (CPR) and the P450 enzyme is the proximal side, and it is mainly triggered by electrostatic interactions between the positively charged surface of P450s and the negatively charged surface of CPR [55,56,57]. For aromatase, many basic residues have been identified and suggested to be involved in the interaction with CPR by site-directed mutagenesis experiments [58] and computational studies [59,60,61]. The conservation of these residues was checked in the multiple alignments, and the results are shown in Table 2. The conservation score is included for each position, together with the result of the visual analysis that allows us to identify the sequences where the amino acids are not conserved.

Out of the nine basic residues that form the positively charged proximal site (Figure S1), six are conserved as their mutation, when present, is conservative. The other three residues appear during evolution at different times, as K352 is conserved in mammals and K389 and K420 are well-conserved starting from amphibians. Concerning the four residues predicted to form hydrogen bonds with CPR, two of them are conserved, and, interestingly, Q351 and Y424 are conserved only in mammals.

These data indicate that a patch of basic amino acids had already appeared in invertebrates, and it has been highly conserved during evolution. However, other residues were introduced later; these comprise the amino acids that reinforce the positively charged proximal site as well as two residues that protrude from the proximal site of the enzyme (Figure S1) to form H-bonds with the redox partner. These data suggest that the interaction with the redox partner has been one of the driving forces for evolution in aromatase.

### 2.2. Homology Modeling of Evolutionarily Old Aromatase

Based on the sequence alignment, homology modeling was applied to two aromatase sequences as it was found that they carry significant insertions, in addition to mutations, in key positions.

The invertebrate aromatase sequence from *Branchiostoma floridae* was selected as it shows an amino acid insertion, 40% of identity, and 60% of homology with the human one. Thus, a homology model was built to study where the main differences between the two aromatase enzymes are located.

**Figure 4:**
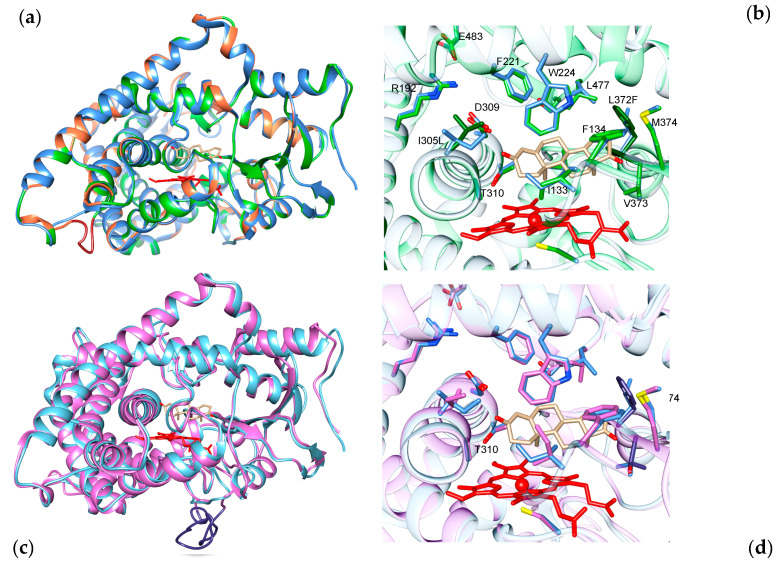
Homology models of evolutionarily old aromatase. (**a**) Homology model for aromatase from the invertebrate *Branchiostoma floridae* (green) superimposed onto the crystal structure of human aromatase (blue). The nonconserved regions are shown in orange, and the grey shadow shows the location of the insertion. (**b**) Zoomed-in view of the active site showing the conserved (green) and nonconserved residues (dark green) involved in substrate binding and catalysis. (**c**) Homology model for aromatase from the pufferfish *Takifugu rubripes* (magenta) superimposed to the crystal structure of human aromatase (blue). The grey shadow shows the location of the long insertion (violet). (**d**) Zoomed-in view of the active site showing the conserved (magenta) and nonconserved residues (dark purple) involved in substrate binding and catalysis. Heme is shown in red and the substrate androstenedione in light brown.

A six-amino-acid insertion is present in the invertebrate sequence compared to all the other sequences analyzed (between M276 and D277 in human aromatase), and the model shows that such an insertion elongates the loop connecting helices H’ and the H loop (Figure 4). Moreover, the analysis of the location of the substitutions shows that they are all on the protein surface and on structural elements such as helix G, which are the least conserved ones in aromatase. There are no mutations in the core structure of the protein and the active site, indicating that the main structural scaffold of aromatase was already present in this old protein. Moreover, many mutations are located in the SRSs, indicating that these areas have evolved in vertebrates.

The multiple sequence alignment also shows the presence of some important mutations together with a long insertion in aromatase from some fish species, including the one from pufferfish *Takifugu rubripes.* In this case, the fish sequence shares 52% of identity and 70% of homology with the human one. A homology model was built in order to predict the possible effect of the substitutions found in the active site. Figure 4 shows the model carrying a long insertion between N421 and V422, which corresponds to the loop connecting helix K’’ and helix L.

Since this long insertion is modeled as a long loop, secondary structure prediction tools were used to verify a possible elongation of the K’’ helix. However, both PsiPred and I-Tasser servers did not predict any secondary structure formation for the amino acids present in that loop. Such a result justifies the absence of such a long and not-necessary loop in the other aromatase sequences.

Concerning the active site, while the substitution of L372 with a phenylalanine does not seem to affect the polarity and dimensions of the catalytic pocket, the substitution of V373 with the polar threonine residue, which in some species is a serine, can be predicted to affect the polarity of the active site (Figure 4). As the substrate carries at least two keto- (as in androstenedione) groups or one keto- group and one hydroxyl group (as in testosterone), the presence of a serine/threonine residue can be predicted to possibly affect the orientation and positioning of the substrate in the active site of the enzyme. Indeed, the Thr/Ser residue could form a hydrogen bond with the substrate. Thus, this substitution seems to be important to properly orient the substrate in the active site for efficient catalysis.

## 3. Discussion

Aromatase is a unique enzyme carrying out a three-step reaction on the androgen substrate, with the third step leading to the aromatization of the A-ring of the steroid molecule. This intriguing reaction has been the subject of many studies aimed at understanding the mechanism of the third aromatization step [62,63]. Moreover, the crystal structure of the human enzyme has indicated the amino acids within the protein matrix involved in substrate binding and catalysis, and their role has been confirmed by site-directed mutagenesis [64,65].

In this work, sequence and structural alignments were performed with aromatase sequences available on databases. Unfortunately, the number of sequences for the different classes of vertebrates is very different as most of the sequences are available from fishes and mammals and, therefore, a bias is introduced in the conservation score. However, we performed a qualitative analysis in order to see the effect of mutations in key positions using the conservation score as an indicator for the level of conservation.

The multiple alignment shows that the enzyme structural scaffold and the key functional residues have been highly conserved during evolution, with only few exceptions in the aromatase sequences from fishes and invertebrates. Thus, the structural core elements of the protein carrying the residues involved in substrate binding are evolutionarily old and this is reasonable as they guarantee the specific function that aromatase has in species conservation. On the other hand, while some SRSs have also been well-conserved during evolution, SRS-3 has shown the lowest level of conservation (15% of the residues are highly conserved). SRS-3 is located on helix G, a flexible element, which, together with helix F and the F-G loop, is known to be involved in the opening and closure of the access channel for the substrate. Interestingly, helix F and the F-G loop are much more conserved as they belong to SRS-2, which shows 40% of the residues to be highly conserved. Out of the conserved residues, we could identify the ones unique to aromatase, thanks to a structural alignment with the other human P450 enzymes. The data show that some conserved and unique amino acids, such as N135 and Y220, are involved in H-bond networks and have a structural role that supports the positioning of the residues involved in substrate binding in the active site.

A lower level of conservation is found in some of the amino acids that form the positively charged proximal side and in some other residues that are involved in the interaction with the redox partner through the formation of H-bonds. This finding is very interesting as CPR is shared between many P450 enzymes within the same organism. Moreover, we have recently demonstrated that human CPR has an effector role as it facilitates substrate binding by stabilizing the aromatase open conformation, which is optimal for substrate access to the active site [42]. Thus, the data suggest that one of the driving forces for evolution has been the optimization of the interface between aromatase and CPR in order to make aromatase more competitive for the same shared redox partner. Such an optimization involves the introduction of positively charged residues as well as amino acids that form H-bonds and facilitate CPR binding, which, in turn, promotes catalysis.

The other interesting finding is the poor conservation of some residues known to be involved in post-translational modifications. Phosphorylation is a rapid way to modulate enzyme activity compared to regulation at the gene level. Aromatase activity is affected by phosphorylation, and some of the residues that can undergo this post-translational modification have been identified [51,52,53,54]. Phosphorylation of S118 has been reported to decrease aromatase activity in human cell lines [54]. The residue S118 is highly conserved in aromatase sequences from vertebrates, together with R115, which forms the consensus sequence for PKA. This consensus is missing in invertebrates and in few fish sequences (3%).

Another consensus sequence for PKA, as well as for PKG, involves S267 and/or Thr268. These residues are not present in fishes, whereas the consensus sequence for PKA is present in amphibians. On the other hand, the consensus for PKG, which includes two or three basic residues, has appeared late during evolution as it is present in only 15% of the mammal aromatase sequences. Interestingly, this consensus sequence includes R264 in human aromatase that is mutated into a Cys or His in some polymorphisms that are also reported to alter aromatase activity when used in combination with polymorphic variants of CPR [66]. Moreover, they have been associated with an increased risk for estrogen-dependent pathologies such as breast cancer and polycystic ovary syndrome [67,68,69,70].

The other residue known to be phosphorylated is Y361, which appears in amphibians but is not fully conserved even within mammals. Aromatase phosphorylation in this position has been associated with tumor progression in breast cancer cell lines [52]. Indeed, short exposure to estradiol was found to increase aromatase activity through phosphorylation of a tyrosine residue (Y361) by c-Src kinase in estrogen-dependent MCF-7 breast cancer epithelial cells. The authors hypothesized the presence of a positive nongenomic autocrine loop between estradiol and aromatase in MCF-7 breast cancer cells [52]. Moreover, it was also demonstrated that estradiol impairs the ability of the tyrosine phosphatase PTP1B to dephosphorylate aromatase, resulting in increased aromatase activity and estrogen production [71]. The multiple sequence alignment shows that the tyrosine residue in position 361, located on helix K, which is one of the most conserved structural elements in aromatase, appears in few fish species, but it is poorly conserved even among mammals, where it is substituted by an asparagine residue, as in most fishes.

Taken together, the results of the conservation of the phosphorylation sites show that evolution has introduced and is still introducing amino acids in key surface positions that can be phosphorylated and consensus sequences in order to modulate aromatase activity. Thus, the need for quickly and locally altering the estrogen concentration in cells seems to be the other driving force for the evolution of this enzyme. This finding is supported by the fact that a rapid regulation of aromatase activity is known to occur in neurons [72,73] and teleost fishes express aromatase only in glial cells, indicating that the ability to synthesize estrogens in neurons has been acquired during evolution [74,75]. In the brain, the acquisition of phosphorylable sites may be explained by the need to modulate estrogen production in higher vertebrate neurons, where rapid changes in estrogen levels, as a consequence of aromatase phosphorylation, have been associated with important physiological and behavioral responses [73].

It is interesting to note that if, on the one hand, the introduction of phosphorylation sites can be evolutionarily beneficial, as in the case of brain aromatase, on the other hand, phosphorylation of residues that increases aromatase activity can strengthen the negative effects of estrogens, as in the case of breast cancer.

In conclusion, this study on aromatase shows that molecular evolution has worked to maintain a high selectivity for a substrate-specific human cytochrome P450 such as aromatase. However, based on the mutations introduced in key sites, it has been observed that evolution has introduced residues that optimize the interaction with the redox partner and phosphorylation sites that give the possibility of rapidly modulating its activity through phosphorylation. It will be interesting to extend the study to other P450s that are highly substrate-selective to understand how molecular evolution has worked for this group of P450s.

## 4. Materials and Methods

### 4.1. Multiple Sequence and Structural Alignments

A total of 365 aromatase sequences from vertebrates and the one available from the cephalochordate *Branchiostoma* were retrieved from the Uniprot database [76] using the ConfSurf server [77] in two different searches. The first one included up to 500 sequences closest to the human aromatase sequence, with at least 40% of identity from the reference database “Clean Uniprot”. The second search was performed by searching for up to 500 sequences that sample the list of homologs to the query that was the sequence of human aromatase. In this case, the minimal percentage of identity was 40%. The sequence was extrapolated from the crystal structure (PDB ID 3S79) so that the server could automatically calculate evolutionary conservation scores and map them on the aromatase structure [78]. These parameters were chosen on the basis that they allowed the retrieval of only aromatase sequences that were manually verified.

Out of the 365 sequences aligned, 66 were from mammals, 8 from birds, 12 from reptiles, 18 from amphibians, 259 from fishes, and 2 from the invertebrates of the genus *Branchiostoma*.

The sequences were aligned through the HMMER algorithm [79] and visualized and analyzed with Jalview software [80]. Position-specific conservation scores were computed using the empirical Bayesian algorithm [81]. The scores were normalized so that the average score for all residues was zero and the standard deviation was one. In aromatase, the lowest score associated with a fully conserved residue was −1.103 (N135), whereas the highest score obtained for a nonconserved residue was +2.844 (E181). The amino acid conservation output, together with the structural conservation from ConSurf server, was checked by visual inspection. Visual inspection is always needed to check correct alignment.

The substrate recognition sites (SRSs) in human aromatase were identified from a structural alignment with the crystal structure of CYP2C8 (PDB ID 2NNJ) [82] performed using the software UCSF Chimera [83]. Indeed, the SRSs were annotated [84] based on the CYP2C family [85].

Structural alignments between aromatase and all the other human P450s were performed using PROMALD3D, a multiple-structure-based alignment refined in combination with sequence constraints [43]. The alignment took into account the crystal structures available and the prediction of secondary structure elements for the unknown structures. Once structurally aligned, the conservation score was assigned using the ConSurf server.

The structural analysis of the conserved amino acids was performed using UCSF Chimera software that was also used for figure preparation [83].

### 4.2. Homology Modeling

Homology models were built using the software Modeller 9.25 [86], I-tasser [87], and the crystal structure of human aromatase (PDB ID 3S79, 3EQM) as a template. The best model was selected according to the Z-DOPE score, with energy minimized using Amberff14SB forcefield [88] and subjected to validation using Molprobity [89], ProSA [90], and QMEAN [91].

The homology model of aromatase from *Branchiostoma floridae* was obtained from Modeller with a Z-DOPE score of −1.0. The validation from the ProSA server showed a Z-score of −9.44 that is within the values of known 3D structures of similar length. The QMEN4 value was −2.89, and the Ramachandran plot showed that 94% were in the favored regions.

The homology model of pufferfish was first obtained from Modeller (Z-DOPE score −1.23). The long insertion was modeled as a long loop, as expected. Thus, a secondary structure prediction was carried out using the PSIPred server [92] and I-Tasser [87]. The validation from the ProSA server showed a Z-score of −7.86, which is within the values of known 3D structures of similar length, whereas the QMEAN4 value was −3.05. The Ramachandran plot showed that 94.57% of the residues were in the favored regions.

## Figures and Tables

**Table 1:** Conservation of the residues involved in substrate binding and catalysis in human aromatase. The scores are normalized so that the average score for all residues is zero and the standard deviation is one. The lowest score represents the most conserved position in a protein. For reference, the lowest score associated with a fully conserved residue was −1.103, whereas the highest score obtained for a nonconserved residue in human aromatase was +2.844.

Residue	Location	Conservation Score	Notes
C437	K″-L helix loop	−1.095	
I305	I-helix	−0.936	L/V only in invertebrate *Branchiostoma*
A306	I-helix	−1.002	T in the mammal *Capra hircus*
D309	I-helix	−1.058	Q in CYP19B1 of the fish *Halichoeres tenuispinis*
T310	I-helix	−1.011	I in the fish *Maylandia zebra*
F221	F-helix	−0.805	
W224	F-helix	−0.896	
I133	B-C loop	−1.038	M in pig aromatase isoform 3
F134	B-C loop	−1.073	
V370	K-helix—β3 loop	−1.001	
L372	K-helix—β3 loop	−0.202	Phe in fishes
V373	K-helix—β3 loop	−0.583	S/ T in most fishes and in CYP19A1 of zebrafish and goldfish
M374	β3	−1.031	
L477	β8–β9 loop	−1.011	
S478	β8–β9 loop	−0.828	A in many sequences, starting from mammals to amphibians. S in fishes.
R192	Helix E	−0.974	C or H in some mammals, birds and fishes, including the two isoforms of zebrafish
E483	β9–β10 loop	−0.761	Conserved in the two isoforms of zebrafish and goldfish

**Table 2:** Conservation of the residues involved in the interaction with the redox partner in human aromatase. The scores are normalized, so that the average score for all residues is zero and the standard deviation is one. The lowest score represents the most conserved position in a protein. For reference, the lowest score associated with a fully conserved residue was −1.103, whereas the highest score obtained for a nonconserved residue in human aromatase was +2.844.

Residue	Conservation Score	Notes
K99	0.287	R in most fishes and *Branchiostoma* *floridae*, not conserved in 1 amphibian, 1 reptile, in 15% of fishes and *Branchiostoma belcheri* (S)
K108	−0.024	Always substituted by R
R145	−0.972	Well conserved
K352	1.293	Conserved only in mammals
K389	0.767	Not conserved in invertebrates (P) and 70% of fishes (including only isoform CYP19A1 in zebrafish)
K390	−0.231	K or R
K420	0.472	Not conserved in two mammals, 20% of fishes (including CYP19B1 of zebrafish) and E in invertebrates
R425	−0.881	Well conserved with some exceptions in fishes and the invertebrates (T)
K440	−0.897	R in invertebrates
S153	−0.533	T in invertebrates and most fishes
Q351	0.799	Conserved in 90% of mammals
Y424	0.308	Conserved in mammals
Y441	−0.553	Conserved in mammals and amphibians, H in 97% of fishes and T in invertebrates

## Data Availability

The data presented in this study are available on request from the corresponding author.

## Supplementary Materials

Supplementary materials can be found at https://www.mdpi.com/1422-0067/22/2/631/s1.
