# Supplementary material for: Molecular and Structural Evolution of Cytochrome P450 Aromatase
Source: Int J Mol Sci. 2021 Jan 10;22(2):631. doi: 10.3390/ijms22020631 (PMC7827799; doi:10.3390/ijms22020631)
Supplement: Supplementary file 1 [file ijms-22-00631-s001.pdf]

International Journal of
Molecular Sciences

MDPI

Article

Molecular and structural evolution of cytochrome P450 aromatase.

Giovanna Di Nardo [1,*], Chao Zhang [1], Anna Giulia Marcelli [1] and Gianfranco Gilardi [1,*]

[1] Department of Life Sciences and Systems Biology; giovanna.dinardo@unito.it

* Correspondence: giovanna.dinardo@unito.it; gianfranco.gilardi@unito.it

Received: date; Accepted: date; Published: date

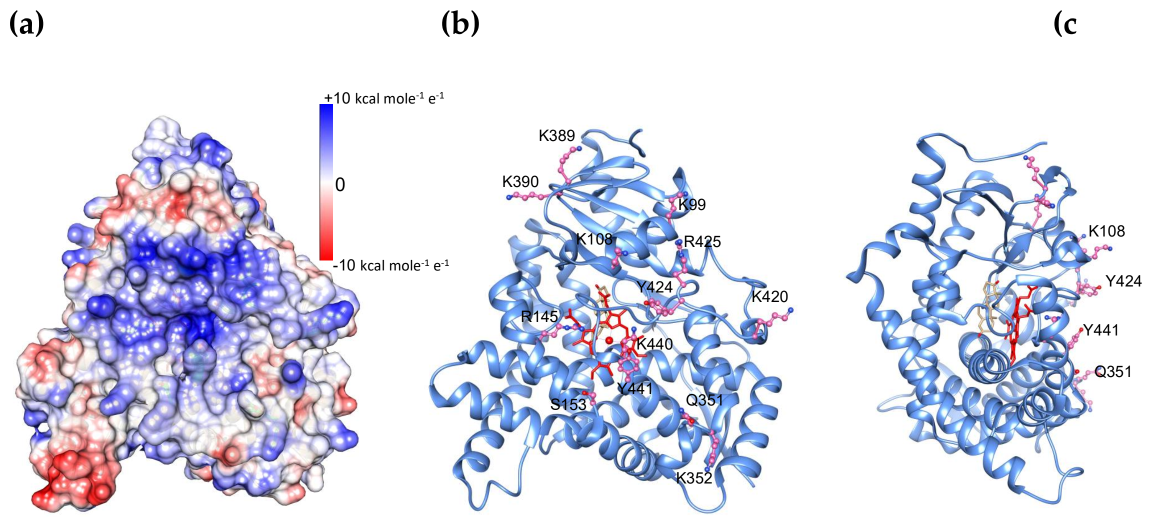

Figure 1. Electrostatic surface of human aromatase and residues important for the interaction with the redox partner cytochrome P450 reductase (CPR). (a) Electrostatic surface of the proximal side of human aromatase. (b). Amino acids involved in the interaction with CPR and (c) residues protruding from the proximal side of aromatase.

Table S1. Percentage of amino acids belonging to the six SRSs conserved in at least 80% of aromatase sequences.

SRS	Location	% of conservation
1	Helices B′-C	42.4
2	Helices F-G′, F-G loop	39.4
3	Helix G	15.4
4	Helix I	69.7
5	Helix K – β1-4	39.1
6	β4-1 – β4-2	42.9

Table 2. Conservation of the residues belonging to the six SRSs in aromatase and in all human cytochromes P450. The scores are normalized, so that the average score for all residues is zero, and the standard deviation is one. The lowest score represents the most conserved position in a protein. In aromatase alignment, the lowest score associated to a fully conserved residue is -1.103 whereas the highest score obtained for a non-conserved residue in human aromatase was +2.844. In the alignment of human P450s, the lowest score is -2.088 and the highest score is +2.865. The residues shown in red represent the ones only conserved in aromatase. The residues shown in green represent the ones conserved in aromatase and present in the other human P450 enzymes.

**SRS1**			**SRS2**		
**Residue**	**Conservation score in aromatase**	**Conservation score in human P450s**	**Residue**	**Conservation score in aromatase**	**Conservation score in human P450s**
LEU120	2.838	-0.454	LEU208	1.012	0.257
GLY121	-1.014	0.382	ASP209	-0.03	0.571
LEU122	-1.015	1.389	GLU210	-0.595	0.574
GLN123	0.365	0.94	SER211	0.919	0.872
CYS124	0.123	0.889	ALA212	0.837	0.76
ILE125	0.343	0.195	ILE213	-0.211	-0.441
GLY126	-0.947	0.166	VAL214	-0.489	0.326
MET127	-1.1	0.325	VAL215	2.78	1.164
HIS128	0.12	0.808	LYS216	-0.902	0.029
GLU129	-0.46	0.737	ILE217	-0.897	0.223
LYS130	0.067	2.175	GLN218	0.932	0.857
GLY131	-1.011	-0.659	GLY219	1.119	-0.099
ILE132	-0.436	-0.184	TYR220	-1.005	-0.342
ILE133	-1.038	1.245	PHE221	-0.805	0.015
PHE134	-1.073	1.365	ASP222	0.532	1.045
ASN135	-1.103	-0.342	ALA223	-0.805	0.748
ASN136	-0.549	0.439	TRP224	-0.896	2.245
ASN137	-0.751	0.435	GLN225	-0.894	0.763
PRO138	0.025	-0.571	ALA226	0.266	0.343
GLU139	2.701	0.508	LEU227	-0.808	0.161
LEU140	0.61	0.577	LEU228	-0.648	0.793
TRP141	-0.773	-1.464	ILE229	-0.583	0.116
LYS142	0.128	0.062	LYS230	-0.528	1.462
THR143	0.509	0.296	PRO231	-0.967	0.25
THR144	1.271	-0.263	ASP232	-0.178	-0.864
ARG145	-0.972	-1.607	ILE233	0.317	1.707
PRO146	1.01	-0.349	PHE234	-0.674	0.133
PHE147	0.303	-0.28	PHE235	-0.816	0.48
PHE148	-0.746	-0.624	LYS236	0.003	1.32
MET149	1.128	0.393	ILE237	0.921	1.591
LYS150	-0.665	0.002	SER238	0.867	0.436
ALA151	-0.932	0.172	TRP239	0.055	0.912
LEU152	-0.952	-1.254	LEU240	-0.009	1.953
**SRS3**			**SRS4**		
**Residue**	**Conservation score in aromatase**	**Conservation score in human P450s**	**Residue**	**Conservation score in aromatase**	**Conservation score in human P450s**
TYR241	0.286	0.209	ARG293	0.48	0.45
LYS242	1.005	0.156	GLU294	0.034	-0.822

LYS243	-0.018	0.502	ASN295	-0.442	-1.144
TYR244	-0.353	1.016	VAL296	-1.102	-0.775
GLU245	0.605	2.504	ASN297	-0.073	1.428
LYS246	1.49	0.737	GLN298	-1.096	0.374
SER247	-0.393	0.081	CYS299	-0.592	-0.371
VAL248	0.079	0.665	ILE300	-0.435	-0.064
LYS249	0.169	0.021	LEU301	-0.878	0.828
ASP250	-0.368	2.607	GLU302	-1.092	-1.067
LEU251	-0.767	-0.047	MET303	-1.031	-0.761
LYS252	-0.452	0.849	LEU304	-0.568	-0.86
ASP253	0.008	0.589	ILE305	-0.936	-0.375
ALA254	0.485	1.172	ALA306	-1.002	-1.644
ILE255	-0.262	-0.364	ALA307	-0.742	-1.285
GLU256	0.733	0.639	PRO308	-1.026	-1.153
VAL257	2.563	1.152	ASP309	-1.058	-1.071
LEU258	-1.075	0.354	THR310	-1.011	-2.046
ILE259	-0.026	-1.173	MET311	-0.306	-1.477
ALA260	0.48	0.289	SER312	-1.02	-1.223
GLU261	1.252	-0.186	VAL313	-0.124	-1.124
LYS262	-1.047	-0.734	SER314	-0.668	-0.945
ARG263	-0.714	-0.578	LEU315	-0.244	-1.232
ARG264	1.207	0.61	PHE316	-0.152	-0.85
ARG265	2.818	0.353	PHE317	-0.879	-1.446
ILE266	0.069	0.357	MET318	-0.811	0.511
			LEU319	-0.892	-0.579
			PHE320	0.55	-0.583
			LEU321	-0.827	0.827
			ILE322	-0.746	-1.486
			ALA323	-0.841	-0.769
			LYS324	-0.417	-0.487
			HIS325	-0.188	-0.494

**SRS3**			**SRS4**		
**Residue**	**Conservation score in aromatase**	**Conservation score in human P450s**	**Residue**	**Conservation score in aromatase**	**Conservation score in human P450s**
LYS354	1.432	-0.695	ILE474	-0.329	2.681
VAL355	-0.114	-1.383	HIS475	-0.812	2.617
MET356	0.604	-1.073	ASP476	-0.101	0.462
GLU357	-0.821	-0.814	LEU477	-1.012	1.058
ASN358	0.159	-1.668	SER478	-0.828	-0.063
PHE359	-0.442	-1.198	LEU479	-0.354	-0.458
ILE360	-1.004	-1.109	HIS480	-0.73	0.159
TYR361	-0.089	-0.976	ILE474	-0.329	2.681
GLU362	-1.05	-2.088	HIS475	-0.812	2.617
SER363	-0.32	-1.056	ASP476	-0.101	0.462
MET364	-0.251	-1.356	LEU477	-1.012	1.058
ARG365	-0.933	-2.088	SER478	-0.828	-0.063
TYR366	-0.248	-1.159	LEU479	-0.354	-0.458
GLN367	-0.827	0.48	HIS480	-0.73	0.159
PRO368	-0.968	-1.01	ILE474	-0.329	2.681
VAL369	-1.001	-0.529			
VAL370	-1.001	-0.506			
ASP371	-0.494	-0.657			

LEU372	-0.202	1.142
VAL373	-0.583	-0.234
MET374	-1.031	-0.123
ARG375	-1.012	-1.876
LYS376	-0.17	2.285

© 2020 by the authors. Submitted for possible open access publication under the terms and conditions of the Creative Commons Attribution (CC BY) license (http://creativecommons.org/licenses/by/4.0/).
